# A Monoclonal Antibody with a High Affinity for Ricin Isoforms D and E Provides Strong Protection against Ricin Poisoning

**DOI:** 10.3390/toxins16100412

**Published:** 2024-09-24

**Authors:** Loïs Lequesne, Julie Dano, Audrey Rouaix, Camille Kropp, Marc Plaisance, Stéphanie Gelhaye, Marie-Lou Lequesne, Paloma Piquet, Arnaud Avril, François Becher, Maria Lucia Orsini Delgado, Stéphanie Simon

**Affiliations:** 1 Département Médicaments et Technologies pour la Santé (DMTS), SPI, Université Paris Saclay, CEA, INRAE, 91191 Gif-sur-Yvette, France; lois.lequesne@cea.fr (L.L.); luorsini@gmail.com (M.L.O.D.); 2 Microbiology and Infectious Diseases Department, French Armed Forces Biomedical Research Institute, 91220 Brétigny-sur-Orge, France

**Keywords:** ricin, ricin isoforms, monoclonal antibodies, neutralizing antibodies, passive immunotherapy, long-term immunity

## Abstract

Ricin is a highly potent toxin that has been used in various attempts at bioterrorism worldwide. Although a vaccine for preventing ricin poisoning (RiVax™) is in clinical development, there are currently no commercially available prophylaxis or treatments for ricin intoxication. Numerous studies have highlighted the potential of passive immunotherapy using anti-ricin monoclonal antibodies (mAbs) and have shown promising results in preclinical models. In this article, we describe the neutralizing and protective efficacy of a new generation of high-affinity anti-ricin mAbs, which bind and neutralize very efficiently both ricin isoforms D and E in vitro through cytotoxicity cell assays. In vivo, protection assay revealed that one of these mAbs (RicE5) conferred over 90% survival in a murine model challenged intranasally with a 5 LD_50_ of ricin and treated by intravenous administration of the mAbs 6 h post-intoxication. Notably, a 35% survival rate was observed even when treatment was administered 24 h post-exposure. Moreover, all surviving mice exhibited long-term immunity to high ricin doses. These findings offer promising results for the clinical development of a therapeutic candidate against ricin intoxication and may also pave the way for novel vaccination strategies against ricin or other toxins.

## 1. Introduction

Ricin is a protein highly toxic for humans and is found in significant amounts in the seeds of the plant *Ricinus communis* (approximately 9 mg/g [1]). The symptoms of ricin poisoning can vary widely, from gastrointestinal or respiratory distress to severe multi-organ failure, depending on the route of exposure [2]. Ricin is a heterodimeric type 2 ribosome-inactivating protein (RIP) comprising an A chain (RTA) and a B chain (RTB), which are linked by a disulfide bond. RTA functions as an rRNA N-glycosylase (EC 3.2.2.22), specifically cleaving an adenine residue from the 28S ribosomal RNA in the 60S ribosomal subunit [3,4]. This cleavage irreversibly inactivates the ribosome, halting protein synthesis and leading to cell death. RTB acts as a galactose-specific lectin, binding to glycolipids and glycoproteins on the cell surface through their terminal galactose or N-acetylgalactosamine residues. Once RTB binds to the cell surface, ricin is internalized and transported retrogradely to the endoplasmic reticulum, where the disulfide bond is reduced. The A chain is then translocated into the cytoplasm, where it exhibits its enzymatic activity. Due to its high toxicity, ease of production, and lack of treatment, ricin is classified by the Centers for Disease Control and Prevention (CDC) as a Category B biological weapon. Its militarization is also banned under both the Chemical Weapons Convention and the Biological Weapons Convention. From the most famous use of ricin as a biological weapon in the assassination of Georgi Markov (1978) [5] to the recent foiled attacks in Cologne and Paris (2018) [6,7], numerous potential uses of ricin as a biological weapon have been reported.

Ricin exists in different variants, with different toxicities reported in vitro and in vivo [8,9,10,11,12]. The toxicity of ricin can be influenced by post-translational modifications, such as N-glycosylation, which can vary depending on the cultivar [8]. Additionally, the ricin sequence can vary as it exists in two main isoforms: D and E [11]. Isoform D is present in all cultivars and, in some cases, like cv. Zanzibariensis, it can be the only isoform found [13]. More often, ricin exists as a mixture of both isoforms D and E, with variations in their relative proportions [9,14]. Ricin D and E differ only in their B chain sequence: the B chain of ricin E is composed of the N-terminal half of ricin D B chain and the C-terminal half of RCA120 B chain, an agglutinin found in *R. communis* seeds, sharing high sequence homology with ricin but being 50 to 2000 times less toxic for humans [10,11,15].

Despite extensive research over the past decades, no clinically approved prophylaxis or treatment for ricin poisoning has yet been approved. The literature offers extensive insights into the development and evaluation of a range of therapeutic approaches, including monoclonal and polyclonal antibodies as well as chemical compounds [16,17,18,19,20,21,22,23,24,25,26,27,28,29,30,31,32]; for review, see [33]. Among all, passive immunotherapy using antibodies remains the most promising post-exposure treatment. Therefore, numerous antibodies targeting ricin have been thoroughly studied, with detailed descriptions of their interactions with either RTA, RTB or the interface between the two. These studies address various aspects, including the identification of the paratope and/or the epitope, the determination of the affinity, and some insight into the mechanisms of neutralization. The latter includes blocking cellular binding, disrupting retrograde transport, redirecting the toxin to degradation compartments, or inhibiting enzymatic activity [30,32,34,35,36,37]. Additionally, research evaluates the neutralization efficiency of these antibodies in cellular models and their protective efficacy in preclinical studies, primarily involving mice and non-human primates [20,27,28,38,39,40,41]. Nevertheless, among all the neutralizing antibodies developed, only a few have been shown to be protective in vivo in post-exposure approaches, which underscores the challenging nature of developing effective ricin countermeasures [42,43,44]. 

While many studies have reported promising results in in vivo preventive protection, often involving the pre-incubation of antibodies with ricin before administration to the mice, only a few focused on post-exposure treatments [20,27,28,38,39,40,41]. Comparing protection levels across studies is challenging due to differences in experimental models, administration routes, ricin doses, and therapeutic windows. However, it is clear that the effectiveness of these antitoxin therapies is influenced by several factors, including the timing of treatment initiation relative to toxin exposure, the antibody’s affinity for the toxin, and the specific mechanisms of neutralization. From a clinical perspective, an extended therapeutic window may be necessary for effective treatment, as therapeutic intervention can often only be implemented long after exposure and after the penetration of the toxin into the cells. Unfortunately, the efficacy of protective antibodies against ricin decreases significantly if they are not administered within hours of exposure [38]. 

Among the antibodies described as effective post-exposure treatments, some are conventional monoclonal IgGs targeting RTA, such as PB10 [29], while others target RTB, like MH75 [38] or the previously published RB34 and RB37 [28]. Engineered single-domain antibodies, such as bispecific VHH antibodies targeting both RTA and RTB, have also demonstrated prophylactic and therapeutic potential in a murine model [45]. Alternatively, combining antibodies with other chemical agents, such as anti-inflammatory drugs, has proven successful in previous studies [46,47].

Because of the specificity of antibody recognition and the existence of ricin variants, designing antibody-based therapeutic solutions efficiently against all ricin mixtures is not straightforward. In fact, information about ricin is often incomplete in studies developing anti-ricin antibodies, especially concerning ricin origin, such as specific cultivars and isoforms. Additionally, it is seldom mentioned whether antibodies described as protective are effective against both isoforms D and E from various cultivars. Antibodies targeting RTA are generally expected to have broad-spectrum recognition, as RTA are nearly identical across cultivars. However, recognizing RTB may be more challenging due to significant variations, particularly in the second half of the C-terminal region. The specificity of antibodies for certain epitopes can offer insights into their potential to recognize different ricin isoforms. Nevertheless, this information needs to be complemented by in vivo studies assessing protection against a mixture of ricin D and E, as such combinations could be encountered in a malicious attack. In the hypothesis of treatments using either an anti-RTB alone or combining an anti-RTA with an anti-RTB to increase protective efficacy, it is crucial to develop broad-spectrum antibodies. Our previous study has demonstrated that the effectiveness of antibody combinations can rapidly decrease if the antibodies are unable to recognize both isoforms [27].

In this article, we developed antibodies that can equivalently recognize both isoforms D and E of ricin and assessed their neutralizing and protective abilities. We selected two antibodies: one targeting RTA (RicE5) and another targeting RTB (RicE8). Although both antibodies exhibited strong neutralizing capabilities in cellular models, only RicE5 provided effective protection in an in vivo intoxication model. Administering RicE5 6 h after ricin intoxication resulted in a survival rate of over 90%, while administration 24 h post-exposure achieved up to a 35% survival rate. Additionally, mice that survived ricin intoxication through passive immunotherapy developed robust long-term immunity to ricin, a finding that may hold promise for future vaccine development.

## 2. Results

### 2.1. Production and Selection of a New Generation of Anti-Ricin Monoclonal Antibodies (mAbs)

As previously described, depending on the *Ricinus communis* cultivar, seeds contain different proportions of D and E isoforms of ricin toxin [1,48,49]. We demonstrated that this could moderately impact ricin-induced toxicity in a cultured cell model. More importantly, it significantly affects the neutralizing (in vitro) and protective (in vivo) abilities of therapeutic antibodies, especially those targeting the B chain, which exhibits peptide sequence differences between the D and E isoforms [27]. Therefore, we have decided to develop a new generation of mAbs capable of binding to and neutralizing both ricin isoforms. To achieve this, mice have been immunized with purified inactivated ricin E isolated from the seeds of *R. communis* cv. Carmencita and those presenting the highest polyclonal antibody immune response specific to ricin D and E were chosen for the fusion of spleen cells with myeloma cells according to the Köhler and Milstein method (Figure S1) [50]. To identify mAbs that recognize identical peptide sequences of ricin D and ricin E, we performed a double-screening ELISA on hybridoma culture supernatants, using biotinylated Ricin D or Ricin E. Based on these results, we selected and cloned eight unique hybridomas using the limiting dilution technique. MAbs were then purified from their culture supernatants and assessed in vitro for their neutralizing capacity.

#### 2.1.1. Screening for In Vitro Neutralizing Capacity of mAbs

**Figure 1:**
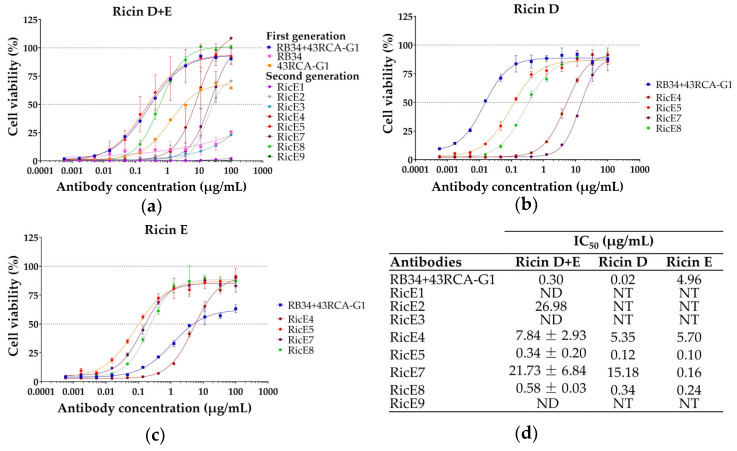
Neutralizing capacity of new generation mAbs on Jurkat cells. Ricin was applied at a final concentration sufficient to induce over 95% of cell death, and mAbs at concentrations varying from 500 pg/mL to 100 µg/mL. (**a**) Neutralization of an equimolar mixture of ricin D and E. Selected anti-ricin mAbs identified in the screening on ricin D + E were evaluated for (**b**) neutralization of ricin D and (**c**) neutralization of ricin E. (**d**) 50% inhibitory concentration (IC_50_) of mAb measured for each test on Jurkat cells. Each point is a mean ± SD of two independent experiments (**a**) (except for RicE1, RicE2, RicE3, and 43RCA-G1 (one experiment)) or of technical replicates from one experiment (**b**,**c**). ND: not detectable, NT: not tested.

The neutralizing capacity of the mAbs was evaluated using a previously optimized cytotoxicity assay [27,28]: Jurkat cells were incubated with a pre-established concentration of ricin inducing more than 95% mortality (D or E, or a 1:1 mixture of D + E from *R. communis* cv. Carmencita), along with mAb concentrations ranging from 500 pg/mL to 100 µg/mL (Figure 1). The 8 mAbs were first assessed for their ability to neutralize an equimolar mixture of ricin D + E and compared to our first-generation mAbs [27] (Figure 1a). The neutralizing capacities of RicE5 and RicE8 are individually equivalent to those of the first-generation mAb combination RB34 + 43RCA-G1. 

Three mAbs, namely RicE2, RicE4, and RicE7, exhibited intermediate neutralizing capacities between 43RCA-G1 and RB34 + 43RCA-G1, while three mAbs (RicE1, RicE3, and RicE9) were unable to neutralize ricin D + E. The four most effective antibodies against the ricin D + E mixture (RicE4, RicE5, RicE7, RicE8) were selected to evaluate their neutralizing capacity against ricin D and ricin E individually (Figure 1b,c) and their half maximal (50%) inhibitory concentration (IC_50_) calculated (Figure 1d).

We observed that RicE5 and RicE8 neutralized both ricin isoforms with comparable efficacy, with RicE5 exhibiting IC_50_ values of 0.12 and 0.10 µg/mL and RicE8 showing IC_50_ values of 0.34 and 0.24 µg/mL, for ricin D and ricin E, respectively. These two mAbs were more effective against ricin E compared to the first-generation combination RB34 + 43RCA-G1, which was significantly more efficient for ricin D neutralization. RicE7 displayed a neutralizing efficacy against ricin E similar to that of the other mAbs. However, its neutralizing capacity against ricin D was lower by two orders of magnitude. Although RicE4 neutralized both ricin isoforms equally, its IC_50_ was 20X and 15X higher than that of RicE5 and RicE8, respectively (Figure 1d). 

Taken together, the neutralization results indicate that the new generation RicE5 and RicE8 mAbs are at least as effective as the best combination of first-generation mAbs (RB34 + 43RCA-G1) in neutralizing the D + E mixture of ricin. Additionally, they exhibit significantly improved neutralization of ricin E compared to RB34 + 43RCA-G1, achieving results comparable to ricin D neutralization.

#### 2.1.2. Binding Kinetics of mAbs to Ricin Isoforms and Antibody Chain Specificity

We determined the affinity of the four pre-selected mAbs to the ricin D and E isoforms. Our results demonstrate that RicE5 and RicE8 exhibit exceptionally high affinity for both isoforms, with equilibrium dissociation constants (K_D_) inferior to 0.1 × 10^−11^ M (see Table 1), outperforming those of the first-generation antibodies. In contrast, RicE4 displays worse affinity (higher K_D_), although its binding is consistent across both isoforms. Notably, for RicE4, RicE5, and RicE8, the differences in affinity are primarily due to variations in dissociation rate constants, while the association rates are relatively similar. Meanwhile, RicE7 demonstrates a very good affinity for ricin E (K_D_ inferior to 0.1 × 10^−11^ M) but shows three orders of magnitude lower affinity for ricin D, consistent with the observed neutralization results. 

**Figure 2:**
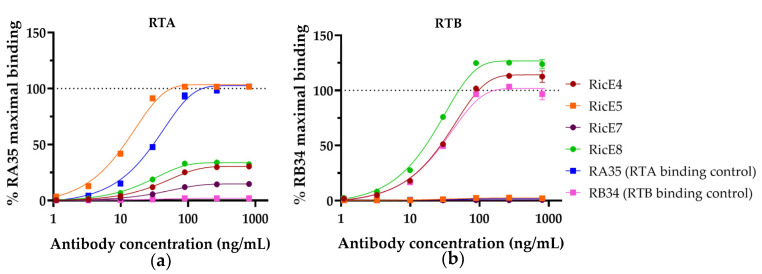
Evaluation of mAb binding specificity to RTA or RTB using sandwich ELISA (**a**) The binding capacities of RicE4, RicE5, RicE7, RicE8, RA35 (anti-RTA antibody control) and RB34 (anti-RTB antibody control) [27,28] to RTA were assessed. The signal is normalized to the maximum binding measured with RA35 for RTA. (**b**) The binding capacities of the same mAbs to RTB were evaluated. The signal is normalized to the maximum binding measured with RB34 for RTB.

To determine whether the mAbs recognize RTA or RTB, we performed a sandwich ELISA using commercially available RTA and RTB. Each antibody was tested at concentrations ranging from 1.4 to 1000 ng/mL with a fixed concentration of biotinylated RTA or RTB in the presence of lactose to avoid nonspecific binding. The results are shown in Figure 2. As controls, we included first-generation mAbs with known chain specificity: RA35, which targets RTA, and RB34, which targets RTB isoform D. Our findings reveal that RicE5 binds specifically to RTA with no significant binding to RTB. Conversely, RicE4 and RicE8 primarily bind to RTB, although they exhibited some binding to RTA, albeit less than RicE5 or RA35. RicE7 demonstrated weak binding to RTA and showed no binding to RTB.

To investigate this further, the purity of both chains was quantified by mass spectrometry, as there was limited information on the commercial ricin chains. This analysis revealed significant contamination of RTA with 3.7% of RTB from ricin D or E (Figure S2). This contamination might account for the weak binding signal of RTA observed with the anti-RTB antibodies. The absence of binding of our anti-RTB control mAb RB34 to the RTA solution (Figure 2a) can be attributed to its specific recognition of the ricin D RTB. Therefore, if RTA is contaminated with the RTB from ricin E or a mixture of ricin D and E, RB34 would either not bind or bind very weakly compared to other antibodies. This hypothesis is supported by the fact that RicE7, which recognizes preferentially ricin E, binds to contaminated RTA (Table 1). Moreover, RicE7 did not bind to commercial RTB (Figure 2b), whereas RB34 did, suggesting that the commercial RTB used was predominantly from ricin D. Altogether, this indicates that RicE7 is primarily an anti-RTB mAb specific to ricin E. 

RicE4 and RicE8 displayed a superior binding capacity to RTB compared to RB34 (Figure 2b). This can be explained by their overall higher affinity for both ricin isoforms compared to first-generation mAbs, as shown in Table 1. The enhanced binding signal to contaminated RTA for these two mAbs, compared to RicE7 or RB34, is likely due to their broader recognition of both ricin isoforms.

Based on the results of the neutralizing capacity and binding kinetics assays, we selected mAb clones for further analysis: one anti-RTA mAb (RicE5) and one anti-RTB mAb (RicE8).

### 2.2. In-Depth Analysis of the In Vitro Neutralizing Capacity of the Two Selected mAbs

#### 2.2.1. In Vitro Neutralizing Capacity of Individual mAbs RicE5 and RicE8 and Their Combinations

**Figure 3:**
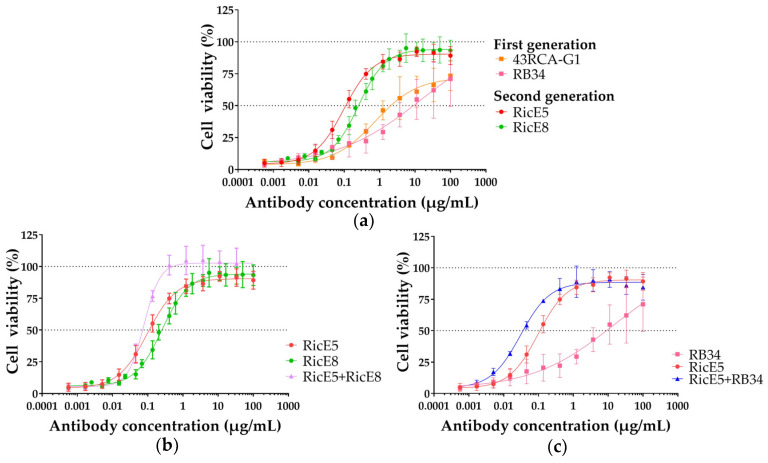
Neutralization curves of mAbs or mAb combinations of an equimolar mixture of ricin D and E on Jurkat cells. Cells were incubated with the ricin mixture at a final concentration of 10 CD_50_ and mAbs at concentrations varying from 500 pg/mL to 100 µg/mL for each experiment. Each point is a mean ± SD of at least three independent experiments. (**a**) Comparison of neutralizing abilities between first-generation and second-generation mAbs. 43RCA-G1, RB34, RicE4, RicE5, RicE8. (**b**) Neutralizing capacities of RicE5 and RicE8 compared to their equimolar combination RicE5 + RicE8. (**c**) Neutralizing capacities of RicE5 and RB34 compared to their equimolar combination RicE5 + RB34.

To evaluate the potential for a synergistic neutralizing effect by combining our mAbs, we tested their neutralizing efficacy in various combinations of two or three with our first-generation mAbs and compared the results to those obtained with each antibody used individually. Jurkat cells were exposed to ricin D, ricin E, or a 1:1 mixture of ricin D and E at a concentration equivalent to 10 times the CD_50_ (mean cytotoxic dose). Additionally, a range of mAb concentrations from 500 pg/mL to 100 µg/mL was tested. Figure 3 shows representative results demonstrating the neutralizing capacity of individual mAbs as well as some of the most effective mAb combinations from the first and second generations. Table 2 provides a summary of the IC_50_ values of the various mAbs and mAb combinations evaluated.

We confirmed that RicE5 and RicE8 demonstrate a significant improvement in ricin neutralization compared to first-generation mAbs, particularly due to their enhanced neutralization of isoform E. For instance, RicE8, which targets the RTB chain, has an IC_50_ value against ricin E significantly lower than that of the first-generation anti-RTB mAb RB34 (*p* < 0.0001). Although some combinations, such as RicE5 + RicE8 and RicE5 + RB34, exhibited lower IC_50_ values, none showed an IC_50_ value significantly lower than RicE5 alone. Furthermore, among the three-mAb combinations, there was no statistically significant difference when compared to the two-mAb combinations or to RicE5 alone.

#### 2.2.2. In Vitro Neutralizing Capacity of RicE5 and RicE8 across Various Cell Lines

To assess the neutralizing capacities of the most promising candidates so far, RicE5 and RicE8, on various cell types beyond Jurkat cells (non-adherent cells derived from a human lymphoblast), we performed in vitro neutralization assays on two epithelial (adherent) cell lines: Vero cells and A549 cells. Vero cells are derived from African green monkey kidney epithelial cells and A549 cells from human alveolar basal epithelial cells. For each cell type, we first determined the CD_50_ values for all ricin types (D, E and a 1:1 mixture of D + E). We observed that epithelial cells exhibited significantly greater resistance to ricin (D, E or D + E) compared to Jurkat cells, with CD_50_ values approximately 4 to 15 times higher in epithelial cells (Table 3). All cell lines demonstrated increased resistance to ricin E compared to ricin D, with Vero cells showing a 6-fold increase, Jurkat cells a 3-fold increase, and A549 cells a 1.5-fold increase. In other words, ricin E seems to be less toxic to these cells than ricin D. The statistical difference between the two epithelial cell lines was not significant for any ricin mixture. The level of cytotoxicity for the 1:1 mixture of ricin D + E was intermediate between the individual isoforms for all cell lines.

**Figure 4:**
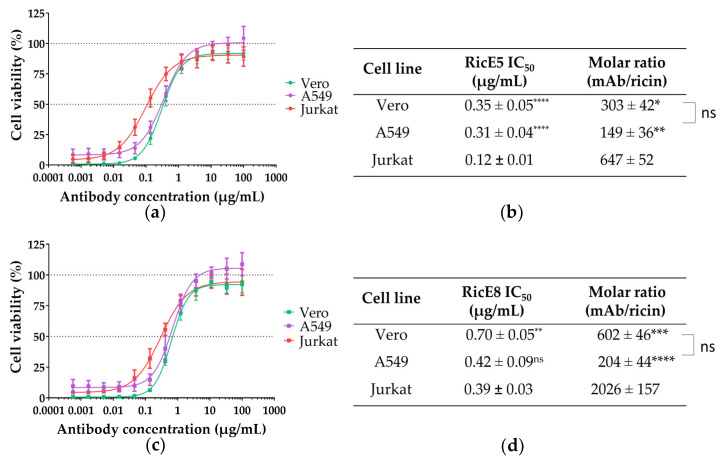
Neutralizing efficacies of RicE5 and RicE8 against ricin D + E in Vero, A549, and Jurkat cells. Each experiment was repeated at least three times. Representative curves of in vitro neutralization of an equimolar ricin D and E solution at 10 CD_50_ by RicE5 (**a**) and RicE8 (**c**). Each point is a mean ± SD. Mean IC_50_ values ± SEM and mAb:ricin molar ratio at the IC_50_ ± SEM of RicE5 (**b**) and RicE8 (**d**). Statistical analysis: One-way ANOVA with Tukey’s post-hoc test. RicE5 or RicE8 IC_50_ values or molar ratios for Jurkat cells, are compared to IC_50_ values or molar ratios for A549 and Vero cells. ns: not statistically significant, *: *p* < 0.05, **: *p* < 0.01, ***: *p* < 0.001, ****: *p* < 0.0001.

For neutralization assays, the 1:1 ricin D + E mixture was used at 10 times the CD_50_ with Ab concentrations ranging from 500 pg/mL to 100 µg/mL. The neutralization curves of RicE5 and RicE8 for A549 and Vero cells are similar (Figure 4a,c). While the IC_50_ values of RicE5 for epithelial cells are higher than for Jurkat cells, this difference is much less pronounced for RicE8. Additionally, the amount of RicE8 required to achieve 50% neutralization of ricin toxicity in Jurkat cells is three times greater than that of RicE5 (molar ratio at IC_50_ of 2026 and 647, respectively). In contrast, the IC_50_ values are fairly similar for A549 cells and differ by a factor of 2 for Vero cells. This variation can be attributed to the different mechanisms of action of the two mAbs (RicE5 being an anti-RTA and RicE8 an anti-RTB) and the differing quantities of RTB receptors among the cell types. 

### 2.3. In Vivo Protective Capacity

We then tested the in vivo protective capacity of the individual mAbs or the mAb combinations with high neutralizing capacity in vitro in a murine model of intranasal ricin intoxication. The fifty percent lethal dose (LD_50_) of an equimolar solution of ricin D + E (1:1) administered intranasally (i.n.) to BALB/cJ female mice was previously determined to be 20 µg/kg using the Miller and Tainter method [27,51].

#### 2.3.1. Treatments 6 h after Intoxication

Six hours after i.n. instillation of ricin D + E at 5 LD_50_ in BALB/cJ female mice, different mAb mixtures at a final dose of 10 mgkg were intravenously (i.v.) injected to evaluate their protective effects.

**Figure 5:**
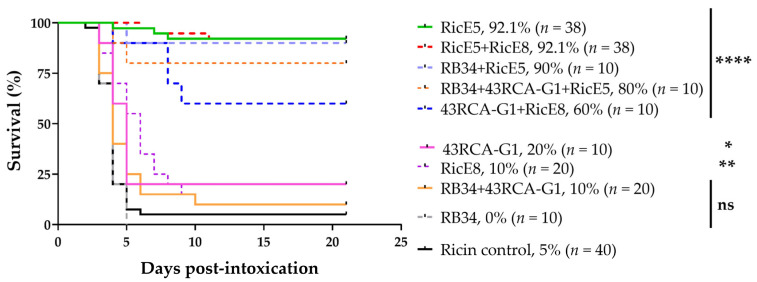
Survival rates of mice treated 6 h post-intoxication. RicE5 and RicE5 + RicE8 were tested in four independent experiments, RicE8 and RB34 + 43RCA-G1 in two independent experiments, and ricin controls in six independent experiments. The other groups were evaluated in a single experiment. The survival rates of the different treatment groups were compared to those of the control group. *n* = number of mice tested in total (8 to 10 mice per group per experiment). Statistical analysis: Log-rank (Mantel-Cox). ns: not statistically significant, *: *p* < 0.05, **: *p* < 0.01, ****: *p* < 0.0001.

As shown in Figure 5, RicE5 alone and in combination with either RicE8 or RB34 provided at least 90% protection. In contrast, the individual first-generation mAb RB34 did not provide any protection. RicE8 and 43RCA-G1 offered only a weak protection (with survival rates of 10% and 20%, respectively, *p* < 0.05), despite RicE8 high affinity for both ricin isoforms and strong in vitro neutralizing abilities. However, a synergistic effect was observed when RicE8 and 43RCA-G1 were combined, significantly increasing the survival rate to 60%. This effect was not observed when the first-generation anti-RTB mAb RB34 was combined with 43RCA-G1, likely due to RB34 specificity for recognizing only the isoform D of RTB. Given the high protection level induced by RicE5 alone (>90%), it is impossible under these experimental conditions to measure the potential contribution of other mAbs associated with RicE5.

#### 2.3.2. Treatments at 10 h, 18 h and 24 h after Intoxication and Addition of Anti-Inflammatory Molecules 

**Figure 6:**
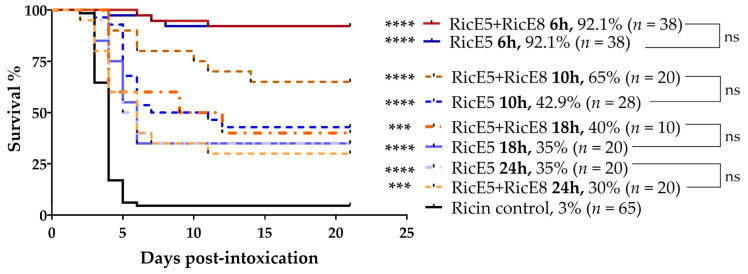
Survival curves of mice treated with RicE5 or RicE5 + RicE8 at 10 mg/kg at various time points (6, 10, 18, and 24 h) following intoxication with 5 LD_50_ of ricin D + E. The data are pooled from multiple experiments (8 to 10 mice per treatment group per experiment); *n* = total number of mice treated per group. For the control group exposed to 5 LD_50_ of ricin, the number of mice per experiment was 5 or 10 mice. Survival rates of the treatment groups were compared with the control group, and comparisons were also made between the different treatments at each time point. Statistical analysis: Log-rank (Mantel-Cox). ns: not statistically significant, ***: *p* < 0.001; ****: *p* < 0.0001. The time of injection post-intoxication is indicated in the legend.

To further challenge RicE5, and with the aim of assessing whether 2-mAb combinations, including RicE5, would provide better protection than RicE5 alone, mAbs were administered at later time points than 6 h after ricin intoxication. When testing RicE5 or RicE5 + RicE8 at different time points (10 h, 18 h, and 24 h post-intoxication), we observed comparable levels of protection with both treatments. RicE5 + RicE8 did not demonstrate statistically significant improvement in protection compared to RicE5 alone at any of the tested time points (Figure 6). These findings indicate that adding RicE8 to RicE5 does not enhance the protective benefits and that RicE5 alone can achieve up to 35% survival when administered 24 h post intoxication. 

Furthermore, previous studies have demonstrated that the molecules ciprofloxacin, doxycycline, and dexamethasone exhibit anti-inflammatory properties and improve protection against ricin at later stages of treatment, especially when combined with anti-ricin antibodies [46,47]. To assess whether adding these molecules to the mAb treatment could enhance protection, mice intoxicated with 5 LD_50_ of ricin D + E were treated with either RicE5 or RicE5 + RicE8 at a dose of 10 mg/kg, along with ciprofloxacin (200 mg/kg) or doxycycline (100 mg/kg) + dexamethasone (4 mg/kg) 24 h post-intoxication. Control groups received either the mAbs or the molecules individually. Our results did not show a statistically significant improvement in survival with the addition of the molecules (Figure S3). 

### 2.4. Induced Long-Term Immunity to Ricin Elicited by Passive Immunotherapy 

Long-term immunity has been previously described as being achieved through passive immunotherapy [22,26,52]. We aimed to evaluate the potential for long-term protection against ricin induced by mAb treatments and to determine if there were differences depending on the treatment received. We hypothesized that the immune complexes formed by the association of mAbs with ricin after their injection might induce immunization in mice that survived an initial ricin exposure due to this passive immunotherapy. 

**Figure 7:**
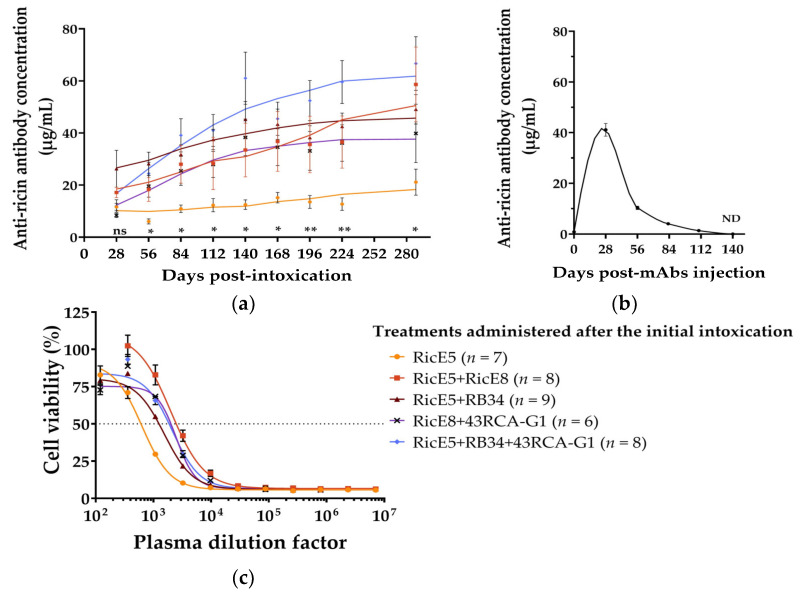
Evaluation of circulating anti-ricin antibodies in mouse plasma. (**a**) Concentration evolution of anti-ricin antibodies in mouse plasma over 300 days. Average concentration (mean ± SEM) of circulating anti-ricin antibodies in the plasma of mice exposed to 5 LD_50_ of ricin treated with 10 mg/kg, 6 h after intoxication, and which subsequently survived (treatments detailed in the legend, *n* represents the number of surviving mice per group). Blood samples were collected monthly for 10 months. Statistical analysis: two-way ANOVA was performed using Tukey’s multiple comparison test. Only the statistics for the comparison of the group treated with one mAb (RicE5, orange line) and the group treated with three mAbs (RicE5 + RB34 + 43RCA-G1, blue line) are represented here. Other comparisons are represented in Figure S4. ns: not statistically significant, *: *p* < 0.05, **: *p* < 0.01. (**b**) Concentration evolution of the RicE5 + RicE8 mixture in mice plasma over 300 days (only quantifiable samples are shown). Plasma samples from 10 individual mice were measured using ELISA. Mice received a single i.v. injection of mAbs (RicE5 + RicE8, 10 mg/kg), and blood samples were collected monthly for 10 months. (**c**) Evaluation of the neutralizing efficacy of an equimolar mixture of ricin D and E at 10 CD_50_ using pooled plasma. The plasma samples evaluated were collected before the second exposure to ricin. Each point is a mean ± SD of at least two technical replicates from one experiment.

To test this, surviving mice were re-exposed to ricin 10 to 12 months after initial exposure and treatment (Table S1). Blood samples were collected monthly following the initial ricin exposure, and the presence of ricin-specific antibodies in plasma was first assessed using ELISA. We observed that the level of circulating anti-ricin antibodies increased over time (Figure 7a). Notably, the amount of circulating anti-ricin antibodies in immunized mice increased with the number of antibodies used for the treatment. Mice treated with three mAbs exhibit significantly higher levels of anti-ricin antibodies over time compared to those that received only RicE5. Given that the half-life of injected murine mAbs in plasma is approximately two weeks [27,28], the measured anti-ricin antibodies are unlikely to be the same as those administered after the initial intoxication. Instead, they are more probably produced by the humoral immune response of the mice. 

To confirm this, a control experiment was conducted in which a group of 10 mice received a single injection of mAbs (RicE5 + RicE8, 10 mg/kg). Blood samples were collected monthly for 10 months, and anti-ricin mAb concentrations were measured using ELISA. Figure 7b demonstrates that the level of circulating anti-ricin mAbs in the plasma of these mice decreased over time, becoming undetectable in less than five months. In addition, these mice were exposed to 5 LD_50_ of ricin ten months after receiving the mAb injection, and none of them survived the challenge. These findings confirm that mice surviving ricin intoxication thanks to mAb treatment developed a robust immune humoral response to ricin, with specific anti-ricin polyclonal antibody concentrations reaching 21 (RicE5 treatment) to 67 µg/mL (3-mAb combination treatment) ten months after exposure. This suggests that the complexes not only circulate for an extended period but also actively elicit an immune response throughout this time.

We then aimed to assess the neutralizing potency of this polyclonal response using the previously described cell-based assay. Plasma samples were collected from mice before re-exposure (i.e., 10 or 12 months after the initial exposure) and pooled by group. These plasma samples were then serially diluted and incubated with Jurkat cells in the presence of 10 CD_50_ concentrations of ricin D + E. The pooled plasma from different groups of mice effectively neutralized ricin, as demonstrated in Figure 7c. Plasma from mice treated with two or three mAbs exhibited an apparently higher neutralizing capacity, as evidenced by the higher dilution required to achieve 50% cell viability (Table 4). However, the antibody concentration in plasma from these mice is also higher compared to plasma from mice treated with one mAb (Figure 7a). Thus, this apparent difference in neutralizing activity is likely only due to a difference in antibody concentration between the plasma pools.

**Figure 8:**
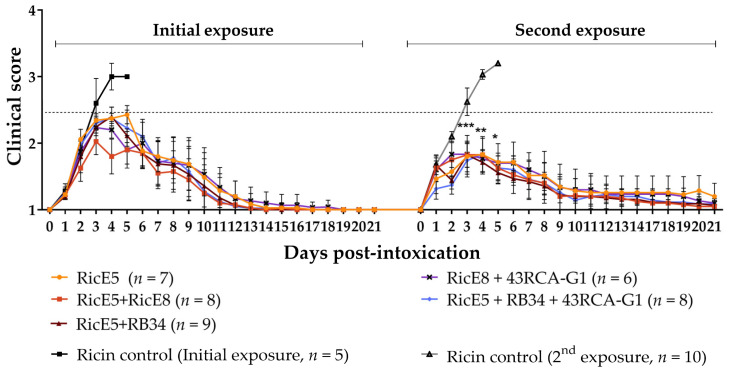
Comparison of clinical signs in mice following initial and second exposure to 5 LD_50_ ricin 10 months apart. Each point of the graph represents the average clinical score of the surviving mice within each group, plotted as the mean ± SD. For the initial exposure, mice received a dose of 5 LD_50_ of ricin D + E, followed by treatment with mAbs at 10 mg/kg 6 h later (or PBS for the “ricin control 5 LD_50_” group). For the second exposure, 10 months after the first exposure, surviving mice were re-exposed to 5 LD_50_ of ricin via the i.n. route with no subsequent treatment. A “ricin control 5 LD_50_” group was included for the second exposure as well. No mice from either control group survived. *n* = number of mice per group that received both exposures. Statistical analysis was performed using a paired *t*-test to compare the maximum clinical scores across all groups on days 3, 4, and 5 after the initial and the second exposure. *: *p* < 0.05, **: *p* < 0.01, ***: *p* < 0.001.

We subsequently re-exposed the mice to the same dose of ricin (5 LD_50_) 10 or 12 months after the initial exposure without administering any additional treatment. All re-exposed mice survived the second ricin challenge, regardless of the treatment received after the first exposure (see Table S1 for a detailed summary of all treatment conditions tested). Additionally, all mice exhibited reduced clinical symptoms during the second ricin intoxication compared to the first one (Figure 8). Despite variations in the antibody titers of the mice plasma, no differences in clinical signs were observed between the different groups, suggesting that protective polyclonal antibodies are present in excess. 

**Figure 9:**
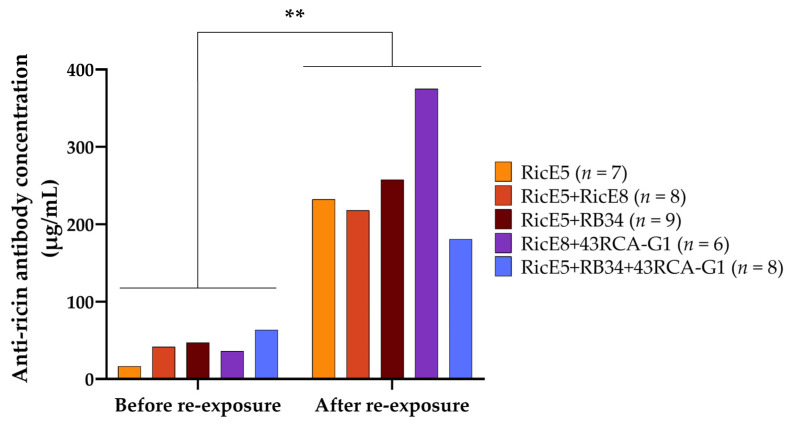
Titration of anti-ricin antibodies in the plasma of mice that survived two exposures to ricin, each at 5 LD_50_, with a 10-month interval between exposures. Plasma samples from mice that received the same treatment after their initial exposure were pooled for analysis (equal volume from each mouse within the same group; details of the groups are provided in the legend, with *n* representing the number of mice per group). Blood samples were collected two weeks prior to the second exposure and 21 days after re-exposure. A paired *t*-test was performed to compare the levels of anti-ricin antibodies before and after the second re-exposure across all groups. **: *p* < 0.01.

When measuring the plasmatic anti-ricin polyclonal antibody levels in mice re-exposed 10 months after their initial exposure, we observed a statistically significant increase in Ab titers, with levels rising up to 10 times higher than those observed at re-exposure (Figure 9). Similar protection was observed in mice re-exposed 12 months after the first ricin exposure (Table S1). The increase in plasmatic Ab concentration translates to a higher neutralizing capacity of the plasma (Table 4). Although RicE8 does not enhance protection in intoxicated mice when combined with RicE5 (as shown in Figure 6), it appears to contribute to the immune response. This is evidenced by the increased levels of neutralizing antibody titers observed with the RicE5 + RicE8 combination compared to RicE5 alone (see Figure 7a,c). Taken together, these results confirm that the long-term protection is attributed to the active immune response generated in mice following ricin exposure and mAb treatment.

## 3. Discussion

Our previous research for protective mAb production resulted in the development of 11 anti-RTA mAbs and 20 anti-RTB mAbs [28]. Among these, the anti-RTB mAb RB34 demonstrated high in vitro neutralizing activity against ricin. It also provided substantial protection to CD1 mice, achieving a 90% survival rate following an intranasal ricin challenge at 5 LD_50_ when administered intravenously one hour after exposure. However, its effectiveness was limited to ricin D [27]. When administering intranasally the same dose of a mixture of both isoforms and concomitantly injecting mAbs intravenously, RB34 only provided significant protection when combined with 43RCA-G1, an anti-RTA recombinant Ab of macaque origin that can recognize both ricin D and ricin E with similar efficacy [27]. The 500-fold difference in the binding affinity of RB34 for ricin E compared to ricin D is likely the reason why this mAb alone cannot protect against a mixture of ricin D + E [27]. To produce protective mAbs targeting both isoforms, we have revised our immunization and hybridoma selection strategies. Our new immunization protocol consisted of using inactivated holotoxin ricin E instead of the previously used RTA and RTB, whose origins were not specified by the manufacturer. In addition, our screening procedure consisted of selecting hybridomas capable of secreting antibodies recognizing both ricin isoforms. These two modifications allowed the selection of novel anti-ricin mAbs with significantly improved binding affinities and neutralizing capacities against both ricin D and E compared to the first-generation mAbs. In this production campaign, eight clones were isolated. Among them, two candidates, RicE5 (anti-RTA mAb) and RicE8 (anti-RTB mAb), showed high affinities for ricin D and E (K_D_ in the picomolar range) and exhibited potent neutralizing activities against both isoforms. Notably, RicE5 and RicE8 improved the IC_50_ value for the ricin D + E mixture by over 10-fold compared to RB34 IC_50_. Both candidates were promising leads for therapeutic applications against ricin poisoning, with RicE5 showing slightly superior neutralizing activity compared to RicE8, although the difference was not statistically significant. Moreover, the neutralizing efficacy of these mAbs in the cytotoxicity cell-based assay closely correlates with their binding affinities for ricin isoforms, indicating that the neutralization primarily depends on the capacities of the mAbs to bind ricin with high affinity.

Given that, in the context of biodefense, the exact composition and origin of ricin used in a potential attack would be unknown and that it appears unrealistic to first identify the specific variant and then adjust the treatment accordingly, it is crucial to be able to neutralize a broad range of ricin variants. Therefore, developing treatments that can address a wide spectrum of ricin variants is essential. 

Ricin D and E are found in varying proportions across different *R. communis* cultivars [49]. Since RicE5 can neutralize both ricin isoforms, it should also be effective against ricin from other cultivars than cultivar Carmencita. This is particularly likely because RicE5 is directed against the A chain, which is identical in sequence across both D and E isoforms and among different cultivars. 

With our new mAb production, we also identified RicE7, a mAb that likely binds to RTB, with a higher affinity for ricin E (at least a 2-fold difference compared to ricin D). This is the first mAb described with a higher specificity for ricin E to our knowledge. Consequently, its neutralizing efficacy for ricin E is comparable to our top-performing mAbs RicE5 and RicE8, demonstrating nearly a two-log difference in neutralization between ricin D and ricin E. This high specificity could be useful for detecting specific ricin isoforms, particularly in in vitro diagnostics or forensics contexts, or for analyzing the composition of unknown ricin mixtures.

RicE5 and RicE8 were evaluated in vivo for their efficacy in protecting mice against intranasally administered ricin at 5 LD_50_. Ricin pathogenesis is complex, involving different cell types with varying sensitivities and accessibility to ricin at different stages of intoxication. The pathogenesis also varies based on the method of ricin inhalation (e.g., aerosol, instillation) and the mouse strain used [53]. Research has shown that maximum depurination in mice occurs 48 h post-intoxication, although this depurination varies among pulmonary cell types [54]. In a CD-1 mouse model using intratracheal instillation, pulmonary macrophages and dendritic cells primarily uptake ricin within the first few hours after exposure while binding to alveolar epithelial cells does not peak until 6 h post-intoxication [55]. By 24 h post-intoxication, endothelial cells become involved, with one-third of these cells being eliminated by 72 h. Therefore, administering treatment within a few hours of ricin exposure could minimize lung damage and significantly improve survival chances, though this may be challenging to achieve in real-world scenarios. Developing therapeutic countermeasures that ensure a satisfactory survival rate, even when treatment is administered at later time points, would then be highly advantageous.

RicE5 demonstrated highly effective protection with over 90% survival when administered 6 h post-ricin challenge, though effectiveness decreased as the therapeutic window was extended. However, RicE5 still allowed a mean survival rate of 35% when administered at 24 h post-exposure. These results are quite promising compared to what has already been published. For example, an anti-RTB mAb, MH77, showed 89% survival when administered at 24 h post-challenge in a study where mice were exposed to a 2 LD_50_ dose, which is lower than the 5 LD_50_ dose used in our model [38]. Additionally, it must be noted that in this study, mice were monitored for 14 days (vs 21 days in our experiment), with mortality still observed in the last days of the experiment. It can be assumed that extending the experimental duration by an additional week could potentially lead to a decrease in the survival rate. 

Despite high affinity and potent neutralizing abilities in vitro, RicE8 did not offer protection in vivo. However, when combined with 43RCA-G1, a synergistic effect was observed, increasing the survival rate to 60%, whereas the survival rate with each Ab alone did not exceed 20%. Nonetheless, neither the combination nor RicE8 alone provided better protection than RicE5 alone.

Since Jurkat cells may not accurately represent the pathophysiology of ricin intoxication in lung epithelium, and to address the observed discrepancy between the effective in vitro neutralization by RicE8 and its lack of efficacy in vivo, we performed neutralization experiments using two epithelial cell lines: Vero cells (African green monkey kidney epithelial cells) and A549 cells (human alveolar basal epithelial cells). To accomplish this, the CD_50_ of each ricin solution was first determined for these two cell lines, and the IC_50_ for the RicE5 and RicE8 mAbs were measured using 10 CD_50_ of the ricin solutions.

A549 and Vero cells exhibited lower sensitivity to ricin D cytotoxicity compared to Jurkat cells, as evidenced by their CD_50_ values. Additionally, Ricin E demonstrated even less cytotoxicity than ricin D. This observation was in agreement with previous research, which demonstrates that ricin E has a lower affinity for galactosyl residues compared to ricin D [56,57]. As expected, the cytotoxicity of the ricin D + E combination was intermediate between that of ricin D and ricin E. The literature has already documented variations in sensitivity for ricin and other type II Ribosome Inactivating Proteins (RIP) among different cell types [57,58,59,60]. These differences can be attributed to two main factors. First, for internalization, ricin binds to glycosylated receptors on the cell surface, recognizing terminal galactosides and N-acetyl-galactosamine moieties [61,62], as well as mannose receptors through its own glycans [63,64]. Due to their differing characteristics, Vero and A549 cells may display different cell surface receptors (or in different proportions), as well as intracellular chaperones involved in retrograde transport, compared to Jurkat cells. These variations can influence the binding of the toxin to the cell surface, its internalization, trafficking, and, ultimately, its effectiveness in reaching the cytosol [65,66]. RicE5, an anti-RTA mAb, likely does not prevent ricin from binding to cells or being internalized. Instead, it may function by affecting the intracellular trafficking of the toxin, preventing the separation of its two chains, blocking the translocation of RTA into the cytosol, or inhibiting its enzymatic activity [36,67,68,69,70]. To support these hypotheses, previous research has identified anti-RTA mAbs that interfere with the retrograde transport of the toxin [34,35]. However, to our knowledge, antibodies that can inhibit the enzymatic activity of ricin in vitro have not been shown to neutralize the toxin effectively in cell-based cytotoxicity assays unless they are expressed as intrabodies [36,68].

Using epithelial cell models that more closely mimic the cells affected by pulmonary poisoning did not provide better predictions than Jurkat cells for the in vivo protection results of the RicE8 mAb. In contrast, neutralization experiments using RicE5 and RicE8 showed that, as evidenced by the antibody/ricin molar ratio, the amount of RicE8 required to neutralize ricin in the Jurkat model was significantly higher compared to the other two cell types. This was also observed, though to a lesser extent, with RicE5. 

Although the three-fold difference in IC_50_ values between RicE5 and RicE8 is not statistically significant, it appears to have a substantial impact on in vivo protection. These findings suggest that while the Jurkat model is not perfect, it is a valuable screening tool before conducting in vivo experiments. 

**Figure 10:**
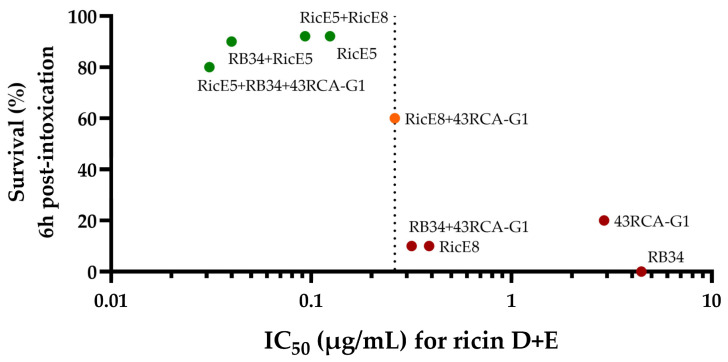
Correlation between the in vivo survival and the IC_50_ of mAb or mAb combinations against ricin D + E isoform mixture (1:1). The survival rate of mice injected with antibodies at 10 mg/kg 6 h post-ricin exposure was plotted against the mean IC_50_ value obtained from in vitro neutralization assays in Jurkat cells. Green: protective mAb or mAb combinations, orange: moderately protective, red: not protective.

Moreover, plotting the survival rate at 6 h against the IC_50_ of our antibodies or their combinations allows us to establish a threshold IC_50_. This threshold, materialized by the IC_50_ of 43RCA-G1 + RicE8, seems to indicate the point above which the antibodies or combinations fail to provide protection (Figure 10). An alternative method could involve assessing protection based on the dissociation constant rate (k_off_) of mAbs, as previously demonstrated [18,36,71]. However, the BLI method used here does not provide clear discrimination in k_off_, and data calculations become more complex when dealing with mAb combinations. 

The IC_50_ threshold could be used in the future to help refine Ab selection before going into animal models, thus helping to reduce animal use and comply with the 3Rs principle (Replacement, Reduction, Refinement). 

Our hybridoma selection process focused on identifying mAbs that recognize both ricin isoforms D and E. This approach may have excluded protective anti-RTB clones that only bind to one isoform. For instance, our first-generation RB34 and RB37 mAbs, which are effective against ricin D but not ricin E, would not have been identified through this type of screening. However, RB37 is noteworthy for its ability to competitively bind to the galactose-binding site, thereby preventing ricin from entering cells. 

In the literature, very few anti-RTB mAbs have demonstrated effective protection in post-exposure scenarios, even when protection is observed, as noted by Noy-porat and colleagues (2017) [38], it is rarely specified whether the antibodies protect against both isoforms. Generally, the ricin preparations used for immunization and screening are not thoroughly characterized or described, and antibody specificities against ricin variants are often not specified or tested. In efforts to develop an effective vaccine against ricin intoxication, Laboratory of N. J. Mantis mapped B cell epitopes on RTB and found that only a small number of antibodies generated following ricin immunizations were truly effective at neutralizing ricin in vitro and in vivo, despite their high affinities. Out of nearly 4000 RTB-specific hybridomas screened, only two anti-RTB mAbs, SylH3 and JB4, were identified as having in vivo protecting properties. These mAbs appeared to bind to similar regions on the RTB surface [37,72]. However, the ricin isoform or cultivar used in these studies is not specified, and some substitutions between ricin D and E are located near the epitopes recognized by SylH3 and JB4, suggesting that these two mAbs might not recognize one of the two isoforms [10]. Additionally, SylH3 was evaluated in a passive protection model where it was either administered prior to ricin exposure or pre-mixed with ricin (of unspecified isoform). Its protective ability in a post-exposure model remains unknown. 

These data raise the question of whether protective antibodies targeting the B chain and recognizing epitopes conserved between the D and E isoforms exist.

As anticipated from the in vitro results, combining RicE5 and RicE8 did not significantly improve survival compared to RicE5 alone when extended therapeutic windows were tested. Similar results were observed by other teams. For instance, a three-antibody combination, including MH77, the aforementioned anti-RTB mAb, provided 80% survival at 24 h post-challenge, which did not improve the survival rate seen with MH77 alone at the same treatment timepoint [38]. However, this antibody cocktail offered protection with a survival rate of 36% even at 72 h post-exposure; a level of survival at such late time points was not achieved by any individual antibody to our knowledge. Moreover, Ab cocktails may offer two advantages: they increase the likelihood of effectively neutralizing natural or intentionally mutated ricin variants by targeting multiple epitopes and may have beneficial effects at the local level, such as reducing pulmonary lesions or inflammatory responses. However, these effects have not yet been evaluated in our model and are not detectable at the clinical sign scale [24]. It is important to note that while RicE8 does not improve survival rate when used in conjunction with RicE5, it does contribute to the long-term immune response induced by the treatment. It cannot, therefore, be ruled out that it may have other additional benefits, such as reducing lesion damage in the lungs.

Improving survival from ricin intoxication may involve mitigating and limiting damage before the complete elimination of the toxin from the body. Preclinical studies in non-human primates and rodents have demonstrated that ricin inhalation triggers acute lung inflammation, often leading to edema that can be fatal [12,73]. Therefore, combining anti-inflammatory molecules with anti-ricin Abs could enhance survival by alleviating symptoms and reducing lung damage, while Abs neutralizes ricin to prevent further inflammation. Gal and colleagues reported that combining Abs with doxycycline and dexamethasone or with ciprofloxacin can significantly improve survival in mouse models of passive immunotherapy against ricin [46,47]. Doxycycline, a tetracycline derivative, has demonstrated anti-inflammatory effects in previous studies involving respiratory tract injuries. Dexamethasone, a potent anti-inflammatory steroid drug, also shows significant promise [47]. Ciprofloxacin, known for its immunomodulatory properties, may help reduce lung inflammation and associated edema [46]. In their model of study, administration of these anti-inflammatory molecules with polyclonal anti-ricin antibodies nearly doubled the survival rate. However, in our study, no significant difference was observed between the use of antibodies alone and antibodies combined with these molecules. It is important to note that the ricin dose they used was lower than the dose administered in our in vivo model (2 LD_50_ vs. 5 LD_50_). This difference may account for the observed discrepancy, as the higher dose of ricin may cause more severe lung damage. Moreover, they used CD-1 mice, while we used BALB/c mice. Given that susceptibility to ricin varies between mouse strains, differences in inflammatory profiles could also exist, potentially contributing to the differing results we observed [53,74]. Despite this, Gal et al. demonstrated that these molecules significantly reduce inflammatory and immune responses in lung injuries [47]. Therefore, their use might still be valuable within shorter therapeutic windows and even before the onset of the symptoms in patients suspected of ricin exposure, potentially improving survival outcomes. 

When ricin is administered via i.n. instillation or aerosol exposure, it primarily accumulates in the upper airways or lungs, with only a small amount entering the bloodstream [75,76]. This minor amount in the blood is negligible compared to the dose in the lungs, where it causes the most severe toxic effects, such as pulmonary edema and respiratory distress. Consequently, protective mAbs administered intravenously are likely to act directly in the lungs rather than capturing ricin in the blood. For optimal and earlier protection, it is crucial that these mAbs rapidly penetrate tissues and reach the organs where ricin accumulates and induces toxicity. Therefore, improving the biodistribution of mAbs and their ability to swiftly penetrate lung tissues could also significantly enhance protective efficacy. Research into antibody formats (such as VHH, F(ab)^’^_2_) and alternative modes of administration (nebulization) may help improve bioavailability in the lungs, enhance deep tissue penetration, and consequently improve the outcome [20,26,77,78].

Literature reported that passive immunotherapy against ricin can elicit a strong, long-lasting humoral response in mice. Reports, though limited, have demonstrated that mice protected from lethal doses of ricin through passive immunotherapy retained high levels of anti-ricin murine IgG in circulation for at least five months [26]. These mice, initially protected by antibody injection, developed a robust humoral response to ricin within ten days after exposure, suggesting that the therapeutic antibodies contributed to the immune response, although independently of the direct protection they provided [26,52]. In our preclinical model, we observed a similar effect with unprecedented time scales. Mice that survived an initial ricin challenge were able to withstand a second challenge at the same lethal dose (5 LD_50_) 10 to 12 months later, regardless of their survival rate after the first exposure. Notably, symptoms during the second exposure were significantly milder compared to the first one. The protective mAbs administered for the initial challenge were cleared from circulation within less than 5 months, and the levels of anti-ricin antibodies produced by the mice increased, reaching nearly 60 µg/mL (the highest concentration) for up to 300 days following the first exposure. No differences in neutralizing potency were observed between the various treatment groups, aside from those attributable to differences in anti-ricin antibody concentrations in the plasma. However, we observed that when an antibody cocktail was used in the direct protection assay, the amount of anti-ricin antibodies in the plasma was notably higher. Furthermore, these levels appeared to increase with the number of mAbs included in the initial treatment cocktail, which, in turn, led to a higher neutralizing titer compared to other treated groups. Based on this observation, we hypothesize that ricin-based immune complexes were formed, likely in the respiratory tract and, more specifically, in the lungs, following the injection of the mAbs for direct protection. These immune complexes may then have led to the activation of the humoral response in mice. This hypothesis aligns with a recent study on mucosal immunization against ricin using ricin-based immune complexes. Tolman et al. showed that a single dose of ricin pre-incubated with a cocktail of two mAbs triggered rapid immunity to lethal doses of ricin. They also observed that using only one mAb to form the immune complex, rather than two, reduced the titer of anti-ricin IgG in circulation without affecting its neutralizing activity. Furthermore, the onset of the humoral response was FcγR-independent and did not directly correlate with the level of inflammation in the lungs. Likewise, ricin immune complexes can induce symptoms in mice after instillation, which do not necessarily result in a higher immune response. They proposed that the activation of the immune system in the respiratory tract may be partly due to the involvement of the complexed ricin, which could promote rapid and efficient uptake by some antigen-presenting cells via mannose receptors, thereby stimulating local T lymphocyte activation [22]. However, Hu et al. [26] observed that the active immunity was not triggered when using F(ab)’_2_ polyclonal antibodies, although these antibodies were as effective as polyclonal IgG in protecting the mice. This suggests that the onset of the humoral response may also be Fc-dependent. Additional data could be generated in our model of re-exposure by using mAbs that lack their Fc. 

## 4. Conclusions

All our findings underscore the critical importance of accurately characterizing protective antibodies against ricin intoxication. They highlight the need to ensure protection against the various isoforms of ricin that could be used in malicious attacks. Such antibodies not only offer direct protection with relatively long therapeutic windows but can also provide robust, long-term protection, even from a single dose following ricin exposure. 

A thorough exploration of ricin preclinical models of passive immunotherapy and the role of immune complexes could pave the way for developing new vaccines or prophylactic measures. These advancements might also be applicable to other toxins, including different type II ribosome-inactivating proteins (RIPs) such as abrin or even to a broader range of respiratory tract pathogens. 

## 5. Materials and Methods

### 5.1. Ethics Statement 

All mouse experiments were conducted in compliance with the French and European regulations on the care of laboratory animals (European directives 2010/63/UE, French Law 2001-486, 6 March 2018) with the agreements of the French Ministry of Higher Education, Research, and Innovation (authorizations APAFIS#3085-2015120909154560v1 and APAFIS#11171-2017090615235646v3), and agreement of the Ethics Committee of the French Alternative Energies and Atomic Energy Commission (CEtEA, no. 044) following the NRC Guide for the Care and Use of Laboratory Animals. All animal experiments minimized suffering in line with the guidelines of the CEtEA committee.

Biozzi mice, bred at the animal care unit of CEA (Gif sur Yvette, France), and BALB/cJ female mice aged 8 to 10 weeks sourced from Janvier Labs (Le Genest-Saint-Isle, France) were housed in ventilated cages, with a controlled environment room maintained at 22 ± 2 °C, with 50 ± 5% humidity and a 12-h dark/light cycle. Biozzi mice were used for immunizations, while BALB/cJ were utilized in protection assays and long-term immunity studies. To ensure acclimatization, mice were allowed to adjust to the housing conditions for one week prior to the beginning of the experiments, as recommended. Food and water were provided ad libitum, and cages were enriched with cellulose mouse huts. For protection assays, HydroGel™ or DietGel™ Recovery (BioServices, Schaijk, The Netherlands) was included in the cages to help prevent dehydration.

### 5.2. Reagents

#### 5.2.1. Ricin Extracts

Ricin isoforms D and E from *R. communis* cv. Carmencita seeds were purified as previously described [27]. The equimolar mixture of ricin isoforms D and E is prepared by combining purified forms of each isoform in equal amounts.

Commercial RTA and RTB were purchased from Sigma-Aldrich (St. Louis, MO, USA). The extent of contamination of RTA by RTB, as well as RTB by RTA, was quantified by mass spectrometry (see Figure S2). Digestion was performed as follows: commercial RTA or RTB at 100 µg/mL in 50 mM ammonium bicarbonate buffer containing 0.05% of RapidGest SF Surfactant (Waters Corp., Milford, MA, USA) was incubated at 95 °C for 15 min. Enzymatic digestion was performed by trypsin (Promega, Madison, WI, USA) at 1 µg/µL at 37 °C for 2 h. Peptides were analyzed by LC-ESI-MS/HRMS (PRM acquisition) using a Q-Exactive Quadrupole-Orbitrap mass spectrometer coupled to an Ultimate 3000 chromatography system (Thermo Fisher Scientific, Bremen, Germany). Peptides were eluted from an Aeris peptide XBC18 reverse phase column (150 mm × 2.1 mm; 1.7 μm; 100 Å; Phenomenex, Le Pecq, France), according to [79]. Quantification was based on specific peptides unique to RTA, RTB, or shared between RTB and RCA120, as previously published in [80].

#### 5.2.2. Preparation of Inactivated Ricin E for Mice Immunization

A 2% paraformaldehyde solution was mixed with purified ricin E at a concentration of 0.5 mg/mL in 0.1 M potassium phosphate buffer pH 7.4 in equal volumes. The mixture was incubated for 48 h at 37 °C. Following incubation, ricin was dialyzed against a 50 mM potassium phosphate buffer, pH 7.4.

#### 5.2.3. Reagents for Enzyme Immuno-Assay (EIA)

EIA buffer, used for sample dilutions and plate saturation, was composed of 0.1 M phosphate buffer, pH 7.4, with 0.15 M NaCl, 0.1% bovine serum albumin, and 1% sodium azide (all from Sigma).

The washing buffer used for the different washing steps was composed of 10 mM potassium phosphate and 0.05% Tween20.

For microtiter plate coating, 96-well Nunc™ MaxiSorp Immuno-Plates (Thermo Fisher Scientific, Illkirch, France) were coated with 100 µL/well of 5 µg/mL goat anti-mouse (GAM) antibodies (AffiniPure™ Goat Anti-Mouse IgG + IgM (H + L) (Jackson Immuno Research Europe Ltd., Ely, UK) and saturated at least overnight at 4 °C with the EIA buffer until use.

Ricin or ricin chains were biotinylated as follows: 1.33 nmol of ricin D, ricin E, RTA, or RTB dissolved in 400 mL of 0.1 M borate buffer at pH 9 was incubated with 13.3 nmol of biotin-N-hydroxysuccinimide ester (Sigma) dissolved in anhydrous DMF. After 30 min at room temperature (RT), 100 µL of 1 M Tris-HCl (pH 8) was added and incubated for an additional 15 min at RT. Finally, EIA buffer was added to a final volume of 500 µL, and preparations were stored frozen at −20 °C until use.

### 5.3. Production of Monoclonal Anti-Ricin Antibodies (mAbs) 

#### 5.3.1. Mice Immunization and Hybridoma Production

Four Biozzi mice were administered inactivated ricin E supplemented with an adjuvant (aluminum hydroxide) via intraperitoneal (i.p.) injection. Each mouse received three doses (5 µg, 20 µg, and 50 µg) of the immunogenic solution at three-week intervals. Prior to the first immunization, blood samples were collected. Subsequent blood samples were taken one week before each immunization to evaluate and monitor the polyclonal anti-ricin D and anti-ricin E response in the sera using a specific Enzyme ImmunoAssay (EIA, see below). The two mice with the highest antibody titers were selected for mAb isolation. Five days before cell fusion, final booster doses were administered to the selected mice. One mouse received 10 µg of native ricin E via intravenous (i.v.) injection while the other mouse received 50 µg of inactivated ricin E i.p., each dose given once a day for three consecutive days. One day after the last booster, both mice were euthanized under anesthesia (isoflurane), and their spleen cells were harvested and pooled. Hybridomas were produced by fusing the harvested spleen cells with NS1 myeloma cells according to the procedure previously described [81]. The culture supernatants were screened for anti-ricin antibodies using an EIA (see below). Selected hybridomas were subsequently cloned by limiting dilution and mAbs purified from the supernatant.

#### 5.3.2. Evaluation of the Polyclonal Response in Mouse Plasma Following Immunization 

Anti-ricin antibodies were detected in the sera of immunized mice or in hybridoma culture supernatants using an EIA. Briefly, 50 µL of serial dilutions of mouse serum in EIA buffer (from 100-fold to 100 000-fold dilutions) or 50 µL of each culture supernatant from 96-well culture plates was transferred into coated microtiter plates. Then, 50 µL of biotinylated ricin D and ricin E (100 ng/mL) was added. After an overnight reaction at 4 °C, the plates were washed, and 100 µL of Acetylcholinesterase (AChE)-labeled streptavidin conjugate (2 Ellman units [EU]/mL) was added to each well. After 1 h incubation at RT followed by five washing cycles, 200 µL of Ellman’s reagent [82] was added, and the absorbance was measured at 414 nm after 30 min and 1 h.

#### 5.3.3. mAb Purification and Recombinant Antibody Production

Mouse mAbs RB34 was purified using high-throughput capture chromatography with Protein A resin (ProSep^®^-A, Merk-Millipore, Darmstadt, Germany). All the mAbs from RicE1 to RicE9 were purified on protein A gel using an HPLC system (AKTA Purifier, GE Healthcare, Uppsala, Sweden). The mAbs were eluted with a 0.1 M glycine buffer at pH 2.5 and dialyzed overnight against a 50 mM potassium phosphate buffer at pH 7.4. Their purity and integrity were determined by polyacrylamide gel electrophoresis (SDS-PAGE), using the Agilent Protein 230 Kit© (Agilent Technologies Inc., Santa Clara, CA, USA) under reducing and non-reducing conditions, according to the manufacturer’s instructions, and their concentration was determined by UV absorbance at 280 nm (Cary 8454 UV-Vis, Agilent Technologies Inc., Santa Clara, CA, USA).

Humanized recombinant Ab 43RCA-G1 was previously produced and purified as described by Respaud et al. [20].

### 5.4. Cell Viability Assays

#### 5.4.1. Cell Growth Conditions

Cell viability assays were performed as previously described with some modifications [27,28]. Briefly, Jurkat cells (from ATCC, Manassas, VA, USA) were cultured in RPMI 1640 without phenol red medium (GIBCO, Grand Island, NY, USA), supplemented with 10% fetal calf serum (GIBCO), 1% glutamine, 1% sodium pyruvate, 1% non-essential amino acid MEM solution and 1% penicillin/streptomycin (all from Sigma), at 37 °C in a humidified atmosphere with 5% CO_2_.

Vero and A549 cells (from ATCC) were cultured in DMEM without phenol red medium (Lonza Walkersville Inc., Walkersville, MD, USA), supplemented with 10% fetal calf serum (GIBCO), 1% glutamine, 1% sodium pyruvate, 1% non-essential amino acid MEM solution, and 1% penicillin/streptomycin, at 37 °C in a humidified atmosphere with 5% CO_2_.

#### 5.4.2. Determination of the 50% Cytotoxic Dose (CD_50_) of Ricin in Various Cell Lines

The protocol used was previously described in [27], with some adaptations. Briefly, for the ricin toxicity tests, various ricin concentrations ranging from 0.12 pg/mL to 250 pg/mL for Jurkat cells and from 0.98 pg/mL to 2 ng/mL for Vero and A549 cells (final concentrations) were distributed in 96-well microplates (Corning Inc., Corning, NY, USA), with cells added at a density of 1000 cells per well (equivalent to 2 × 10^4^ cells/mL). Cells were incubated at 37 °C for 72 h in a humidified atmosphere containing 5% CO_2_. After incubation, cell viability was assessed by indirect measurement of ATP concentrations using the CellTiterGlo^®^ Luminescent Cell Viability Assay kit (Promega, Madison, WI, USA), following the manufacturer’s instructions. Samples without ricin (cells only) were used as controls to determine the 100% viability, allowing a calculation of the percentage viability for each sample.

#### 5.4.3. Evaluation of Antibody Neutralizing Efficacy against Ricin

In the neutralizing activity screening of RicE1 to RicE9 on Jurkat cells (see Figure 1), the ricin concentrations used were based on the lowest concentration, which resulted in over 95% cell death [27]. Specifically, the concentrations were 149 pg/mL for the equimolar mixture of ricin D and E (ricin D + E), 65 pg/mL for ricin D, and 142 pg/mL for ricin E.

To characterize the neutralizing capacities of the mAb or mAb combinations more accurately, we refined and standardized our protocol by using a ricin solution at ten times the 50% cytotoxic dose (10 CD_50_). For Jurkat cells, the 10 CD_50_ values were set at 74 pg/mL for ricin D, 128 pg/mL for ricin E, and 83 pg/mL for ricin D + E. For Vero and A549 cells, the concentrations were 502 pg/mL and 897 pg/mL, respectively, for ricin D + E mixture. 

For each assay, purified mAbs (ranging from 0.56 ng/mL to 100 µg/mL final concentration) were mixed with the toxin in a total volume of 50 µL and pre-incubated for 60 min at 37 °C in 96-well clear flat-bottom polystyrene tissue culture-treated microplates (Corning Inc.). Following pre-incubation, 50 µL of Jurkat, A549, or Vero cells (at a density of 2 × 10^4^ cells/mL, i.e., 1000 cells per well) was added to each well. After 72 h of incubation at 37 °C with 5% CO_2_, cell viability was assessed using the CellTiterGlo^®^ Luminescent Cell Viability Assay kit (Promega) as described above (5.4.2). Wells containing cells without ricin were used as controls to determine the 100% viability, allowing for the calculation of percentage viability for each sample. The effects of neutralizing antibodies were evaluated by testing combinations of two or three antibodies in equal proportions and at concentrations equivalent to those used for single mAbs (0.56 ng/mL to 100 µg/mL total final concentration).

### 5.5. Bio-Layer Interferometry Measurements

The kinetic parameters of the mAbs for the different ricin isoforms were determined using biolayer interferometry on the Octet^®^ Red96e from the fortéBio^®^ system (Sartorius, Fremont, CA, USA), following the manufacturer’s instructions. Briefly, anti-mouse (AMC) or anti-human (AHC) Fc capture biosensors from fortéBio^®^ (Sartorius, Fremont, CA, USA), previously hydrated by immersion in an assay buffer (0.1 M phosphate buffer, pH 7.4, 0.15 M NaCl, 0.1% bovine serum albumin, 1% sodium azide, and 0.02% Tween20) for at least 10 min, were loaded with the mAb of interest by immersion in a solution containing 2.5 µg/mL of 43RCA-G1, and 5 or 10 µg/mL for every other mAbs, for 300 s. Baseline signals were then established by immersing the sensors in the assay buffer for 200 s. The loaded sensors were immersed for 900 s in various solutions containing different concentrations of ricin (one sensor was used for each concentration of the range). For the evaluation of the binding of RB34, concentrations of ricin E ranged from 0 to 80 nM and from 0 to 10 nM for ricin D. For the evaluation of the binding of every other antibody against both ricin isoforms, ricin concentrations ranged from 0 to 10 nM. Changes in the signal due to the association of the ricin with the mAbs were recorded. The sensors were then washed in the assay buffer for 1800 s, and changes in the signal due to the dissociation of ricin from the mAbs were recorded. Data were analyzed using the Octet^®^ Data Analysis HT 10.0 software (fortéBio^®^ LLC, Sartorius, Fremont, CA, USA) with a 1:1 binding model, yielding the association rate constant (k_on_), dissociation rate constant (k_off_), and equilibrium dissociation constant (K_D_).

### 5.6. Evaluation of Ricin-Chain Binding Specificity by Enzyme Immuno-Assay (EIA)

Solutions of mAbs RicE4, RicE5, RicE7, RicE8, RA35, and RB34 at concentrations ranging from 1.1 ng/mL to 800 ng/mL in EIA buffer were added to the wells (100 µL/well) of coated microtiter plates and incubated overnight at 4 °C. After a 3-cycle wash, 100 µL of a 100 ng/mL biotinylated RTA or RTB solution in EIA buffer, supplemented with 100 mM lactose, was added to each well and incubated for 3 h at 21 °C with shaking at 750 rpm. After a 3-cycle wash, 100 µL/well of streptavidin-linked poly-horseradish Peroxidase (Streptavidin Poly-HRP Pierce™, ThermoScientific™, Illkirch, France) diluted 30,000 times (100 times in SeramunStab^®^ for HRP stabilization and 300 times in EIA buffer without azide) was added to each well and incubated for 30 min at 21 °C and agitated at 750 rpm. After a 3-cycle wash, 100 µL/well of TMB (3,3′,5,5′-Tetramethylbenzidine, Sigma) was added to each well and incubated for 30 min at 21 °C in the dark with shaking. Subsequently, 100 µL/well of 2 N H_2_SO_4_ was added to stop the reaction. Absorbances at 450 and 620 nm were measured using a microplate absorbance reader (Epoch Biotek, Gen5 software V 1.09.8). The final optical density for each well was calculated as the difference in absorbance between 450 and 620 nm. Results are represented as a percentage of the maximum binding achieved by the control antibodies for each chain (RA35 for RTA and RB34 for RTB).

### 5.7. Animal Experiments

#### 5.7.1. Passive Immunotherapy Protection Experiments

The fifty percent lethal dose (LD_50_) for intranasal intoxication of mice with an equimolar solution of ricin D and E was previously determined to be 20 µg/kg [27]. Mice (*n* = 10 per group) intranasally exposed to 5 LD_50_ of ricin D + E (i.e., 100 µg/kg) in a maximum volume of 1 mL/kg were treated 6 h post-intoxication with i.v. administration of 10 mg/kg of various mAbs: RB34, 43RCA-G1, RicE5, RicE8. Additionally, combinations of two mAbs (each at a 1:1 ratio, one targeting RTA, the other RTB) were tested: RB34 + 43RCA-G1, RB34 + RicE5, RicE5 + RicE8, 43RCA-G1 + RicE8. A combination of three mAbs (each at a 1:1:1 ratio) was also evaluated: RB34 + 43RCA-G1 + RicE5. A PBS control group was included. Mice were monitored twice a day for 21 days, and clinical signs and survival were recorded. The clinical score was calculated as the average of distress and pain signs: hair score, back score, activity score, and weight loss score. A humane endpoint was applied based on ethics committee recommendations, leading to euthanasia when necessary.

For mAbs demonstrating effective protection at 6 h post-intoxication (RicE5 and RicE5 + RicE8), treatments at later time points (10 h, 18 h, or 24 h post-intoxication) were also evaluated. Furthermore, the co-administration of molecules previously described to have an anti-inflammatory effect [46,47] was evaluated in conjunction with mAb injections and was administered 24 h post-intoxication (Figure S3). The molecules tested included ciprofloxacin-Teva (200 mg/kg, administered in a volume of 10 mL/kg, by i.p. route), doxycycline-hyclate (100 mg/kg, in a volume of 10 mL/kg, by i.p. route), and dexamethasone (4 mg/kg, in a volume of 1 mL/kg, by i.n. route).

#### 5.7.2. Evaluation of Active Immune Response Induction

To assess the induction of an active immune response, mice that had survived the passive immunotherapy protocol were re-exposed to ricin via the i.n. route (as previously described) using the same dose after ten to twelve months without receiving additional treatment (see details in Table S1). Body weight and clinical signs were monitored monthly between the two ricin exposures, and blood samples were collected concurrently to evaluate the presence of anti-ricin polyclonal antibodies (using EIA, see Section 5.8.1) and their neutralizing capacities (via a cell viability assay, see Section 5.8.3). A group of naive mice intoxicated at 5 LD_50_ served as a control for ricin intoxication.

Blood samples were collected monthly for mice that survived ricin intoxication through mAb treatment. Sample collection began 21 days after initial ricin intoxication or mAb injection and continued until mice were re-exposed to ricin, which varied between 10 and 12 months, depending on the experiment (see Table S1). Blood was collected in tubes containing 100 mM EDTA. After centrifugation (10 min, 4 °C, 5000× *g*), the plasma was harvested and stored at −20 °C until use.

As a control, 10 BALB/cJ mice were intravenously administered a combination of RicE5 and RicE8 at a total antibody concentration of 10 mg/kg, the same dose used in the protection experiments. Blood samples were collected monthly for 10 months, and anti-ricin antibody levels were measured by EIA to see how long they were detectable (see Section 5.8.2). After 10 months, the mice were exposed to 5 LD_50_ of ricin i.n., as described above, without any additional treatment.

Following ricin exposure, mice were observed twice daily for 21 days, and clinical signs and survival were recorded. Humane endpoints were applied as needed, based on recommendations from the ethics committee, and euthanasia was performed when required.

### 5.8. Characterization of the Induced Long-Term Immunity against Ricin in Mice

#### 5.8.1. Evaluation of the Polyclonal Response against Ricin in Murine Plasma by EIA

To further characterize the induced long-term immunity to ricin, EIA was performed to quantify the circulating anti-ricin antibodies in the plasma of treated mice. Blood samples were collected as indicated above (Section 5.7.2).

Each plasma sample was diluted 30,000 and 100,000 times in EIA buffer supplemented with 100 mM of lactose. Various concentrations of an anti-ricin- mAb mixture (consisting of RA35 [28], RicE4, RicE5, and RicE8 in a 1:1:1:1 ratio) were diluted in 30,000-fold diluted plasma from naive mice to generate a standard curve ranging from 0.046 to 6 ng/mL (total concentration). These samples were added to microtiter-coated plates (100 µL per well) and incubated overnight at 4 °C. After a 5-cycle wash, 100 µL of an equimolar biotinylated ricin D and E mixture at 50 ng/mL was added to each well and incubated for 2 h at 21 °C with agitation at 750 rpm. Revelation with streptavidin-polyHRP was performed as described in Section 5.6.

#### 5.8.2. Pharmacokinetics of mAb RicE5 + RicE8 Combination

The concentrations of circulating anti-ricin mAb combination in mice plasma were measured as described above (Section 5.8.1), with some minor adaptations. Plasma samples were diluted 30,000 times only in EIA buffer supplemented with 100 mM lactose. The standard curve was generated from a combination of RicE5 and RicE8 mAbs spiked in 30,000-fold diluted plasma from naive mice at concentrations ranging from 7.8 pg/mL to 16 ng/mL (total concentration).

#### 5.8.3. Evaluation of the Neutralizing Capacity of the Murine Plasma against Ricin

The evaluation of the neutralizing capacity of anti-ricin polyclonal antibodies contained in the plasma of immune mice was carried out with some modifications to the protocol described in Section 5.4.3. Plasma from mice belonging to the same treatment group was pooled (using an equal volume from each mouse). This pooled plasma was then diluted 10-fold across twelve dilution points, ranging from undiluted plasma down to a 10^11^-fold dilution. These dilutions were pre-incubated with ricin D + E at 10 CD_50_ (83 pg/mL) for one hour before being applied to Jurkat cells. Cell viability was assessed 72 h later, as described earlier. Note that the graphs do not display cell viability data for the lowest dilution factors due to cytotoxicity from the concentrated murine plasma.

### 5.9. Statistical Analysis

All cell viability assays and in vivo treatment assays were performed at least twice unless otherwise specified. Statistical analyses were performed using PRISM^®^ software v.9.5.1. (GraphPad Software Inc., San Diego, CA, USA). For EIA standard curves, a non-linear regression with a one-phase decay was applied. For cell viability assays, a robust fit using non-linear regression with a four-parameter variable slope was applied. The statistical analysis of survival curves from in vivo experiments was carried out using log-rank (Mantel-Cox) tests.

## Figures and Tables

**Table 1:** Kinetic parameters of neutralizing antibody interactions with ricin D and ricin E using Bio-Layer Interferometry.

Generation	Antibody	Ricin Isoform	K_D_ (M) × 10^−11 #^	k_on_ (M^−1^s^−1^) × 10^5 #^	k_off_ (s^−1^) × 10^−5 #^
1st generation	RB34 ^†^	Ricin D	1.0 ± <0.1	5.61 ± <0.01	0.07 ± 0.01
		Ricin E	542.0 ± 44.3	17.00 ± 0.31	330.00 ± 1.36
	43RCA-G1 ^†^	Ricin D	4.8 ± <0.1	4.23 ± <0.01	2.00 ± <0.01
		Ricin E	9.6 ± <0.1	4.69 ± <0.01	4.52 ± <0.01
2nd generation	RicE4	Ricin D	6.7 ± <0.1	7.70 ± 0.01	5.18 ± <0.01
		Ricin E	13.0 ± <0.1	4.98 ± 0.01	6.45 ± <0.01
	RicE5	Ricin D	<0.1	4.72 ± <0.01	<0.01
		Ricin E	<0.1	3.35 ± <0.01	<0.01
	RicE7	Ricin D	11.8 ± <0.1	13.67 ± 0.03	16.10 ± 0.04
		Ricin E	<0.1	8.22 ± 0.01	<0.01
	RicE8	Ricin D	<0.1 ± <0.1	5.04 ± <0.01	0.03 ± <0.01
		Ricin E	<0.1	3.17 ± <0.01	<0.01

^#^ Values determined by the Octet^®^ Data Analysis HT 10.0 software using a 1:1 binding model and expressed as mean ± SEM. ^†^ Data published in [27].

**Table 2:** Half maximal inhibitory concentration (IC_50_) values for Jurkat cells with a ricin dose corresponding to 10 CD_50_ of ricin D, ricin E, and an equimolar mixture of D + E. Data are represented as mean ± SEM. Statistical analysis: Two-way ANOVA with Tukey’s multiple comparison test by ricin mixture. For each ricin mixture, the statistics of the comparison between the IC_50_ of RicE5 and the IC_50_ of each of the other antibodies or combinations of antibodies are shown. ^ns^: not statistically significant, **: *p* < 0.01, ****: *p* < 0.0001.

	IC_50_ (µg/mL)		
Antibodies	Ricin D	Ricin E	Ricin D + E
RB34	0.03 ± 0.06 ^ns^	16.80 ± 1.52 ****	4.45 ± 0.53 ****
43RCA-G1	2.38 ± 0.46 **	2.47 ± 0.40 ****	2.90 ± 0.37 ****
RicE5	0.14 ± 0.01	0.10 ± 0.01	0.12 ± 0.01
RicE8	0.46 ± 0.04 ^ns^	0.25 ± 0.02 ^ns^	0.39 ± 0.03 ^ns^
RicE5 + RicE8	0.10 ± 0.01 ^ns^	0.09 ± <0.01 ^ns^	0.09 ± 0.01 ^ns^
RicE5 + RB34	0.03 ± 0.02 ^ns^	0.18 ± 0.08 ^ns^	0.04 ± <0.01 ^ns^
RicE8 + 43RCA-G1	0.21 ± 0.02 ^ns^	0.19 ± 0.01 ^ns^	0.26 ± 0.05 ^ns^
RB34 + 43RCA-G1	0.02 ± <0.01 ^ns^	4.78 ± 1.64 ****	0.32 ± 0.04 ^ns^
RicE5 + RicE8 + RB34	0.11	0.17	0.06 ± 0.01 ^ns^
RicE5 + RB34 + 43RCA-G1	0.02 ± <0.01 ^ns^	0.12 ± 0.01 ^ns^	0.03 ± <0.01 ^ns^
RicE8 + RB34 + 43RCA-G1	0.09 ± 0.04 ^ns^	0.31 ± 0.03 ^ns^	0.09 ± 0.03 ^ns^

**Table 3:** Table of 50% cytotoxic dose (CD_50_) measured for ricin D, ricin E, and equimolar ricin D and E on Vero, A549, and Jurkat cells.

	CD_50_ (pg/mL)		
Cell Type	Ricin D	Ricin E	Ricin D + E
Vero	24.9 ± 2.1 ****	157.8 ± 15.0 ****	47.3 ± 3.9 ****
A549	82.4 ± 4.7 ****	126.8 ± 11.5 ****	79.0 ± 7.0 ****
Jurkat	5.6 ± 0.4	17.7 ± 1.6	9.7 ± 0.6

CD_50_ values for each cell type were determined in at least four independent experiments. CD_50_ values are presented as means ± SEM. Statistical analysis: two-way ANOVA and Tukey’s multiple comparison test. Statistics are shown between the Jurkat CD_50_ and the CD_50_ of the other two epithelial cell lines for each ricin solution. ****: *p* < 0.0001.

**Table 4:** Dilution factors of the pooled plasma were calculated to determine the concentration needed to achieve 50% of Jurkat cell viability in the presence of 10 CD_50_ of ricin.

	Dilution Factor for 50% of Viability	
Treatments after Initial Exposure	Before Re-Exposure	After Re-Exposure
RicE5	672 ± 85	2329 ± 256 *
RicE5 + RicE8	2616 ± 378	2710 ± 172 ^ns^
RicE5 + RB34	1265 ± 49	2692 ± 337 ^ns^
RicE8 + 43RCA-G1	1907 ± 136	11,142 ± 1441 ****
RicE5 + RB34 + 43RCA-G1	1790 ± 158	2784 ± 93 ^ns^

Data is presented as mean ± SD from at least two technical replicates within a single experiment. Statistical analysis was conducted using a two-way ANOVA followed by Sidak’s multiple comparison test to compare dilution factors before and after re-exposure across different groups. ns: not statistically significant, *: *p* < 0.05, ****: *p* < 0.0001.

## Data Availability

The original contributions presented in the study are included in the article/Supplementary Materials; further inquiries can be directed to the corresponding author.

## Supplementary Materials

The following supporting information can be downloaded at: https://www.mdpi.com/article/10.3390/toxins16100412/s1, Figure S1. Dosage of anti-ricin antibodies of plasma of mice that were immunized with inactivated ricin E and selected for cell fusion and hybridoma production; Figure S2. LC-MS/HRMS signals of peptides from (a) RTA, (b) RTB, and (c) RTB/RCA120 B-chain in commercial RTA or RTB solutions; Figure S3: Survival curves of mice intoxicated with 5 LD_50_ of ricin D + E and subsequently treated 24 h later with 10 mg/kg of mAbs along with anti-inflammatory molecules; Figure S4. Concentration of anti-ricin polyclonal antibodies in mice plasma after first ricin exposure; Table S1. Summary of the conditions tested for re-exposition of immune mice.
