# Supplementary material for: A Monoclonal Antibody with a High Affinity for Ricin Isoforms D and E Provides Strong Protection against Ricin Poisoning
Source: Toxins (Basel). 2024 Sep 24;16(10):412. doi: 10.3390/toxins16100412 (PMC11510859; doi:10.3390/toxins16100412)
Supplement: Supplementary file 1 [file toxins-16-00412-s001.zip › toxins-3220911-supplementary-need update 9.29.pdf]

# Supplementary Materials: A Monoclonal Antibody with a High Affinity for Ricin Isoforms D and E Provides Strong Protection against Ricin Poisoning

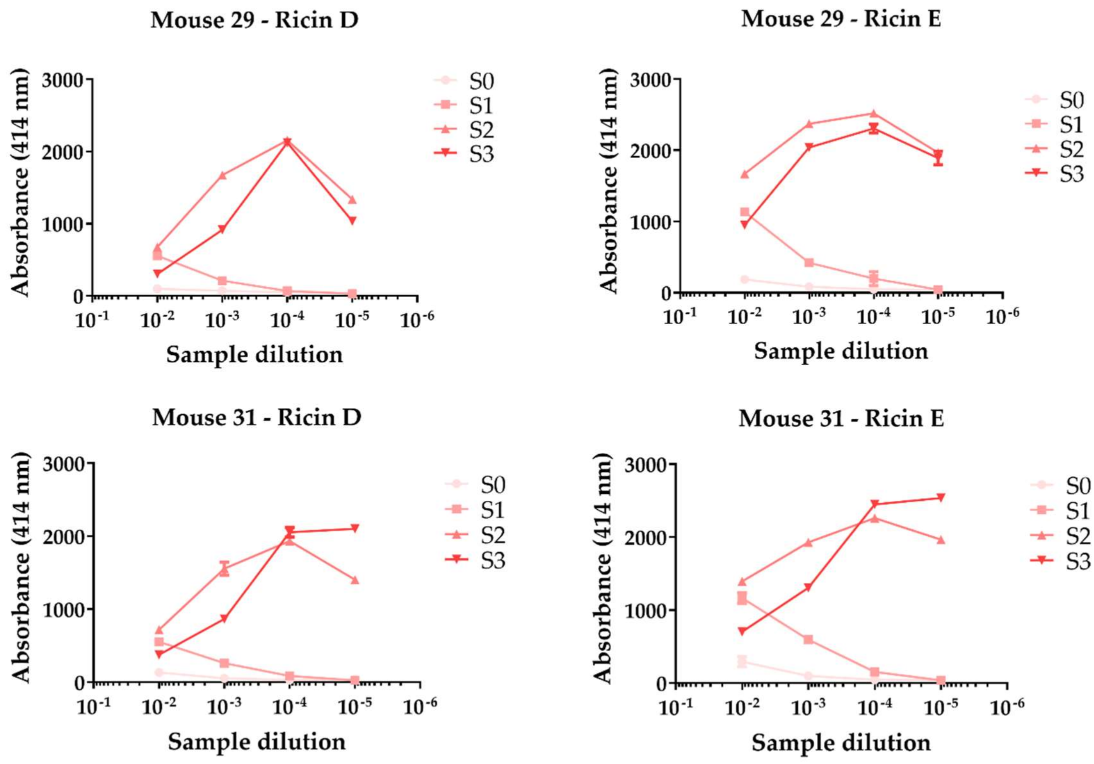


**Figure S1**. Dosage of anti-ricin antibodies of plasma of mice that were immunized with inactivated ricin E and selected for cell fusion and hybridoma production. Blood sample S0 was collected before the first injection of inactivated ricin E and S1 to S2 one week prior a new injection. S3 was collected one week before dosage and two weeks after last immunization. Plasma were quantified for either anti-ricin D antibodies (left column), either anti-ricin E antibodies (right column).

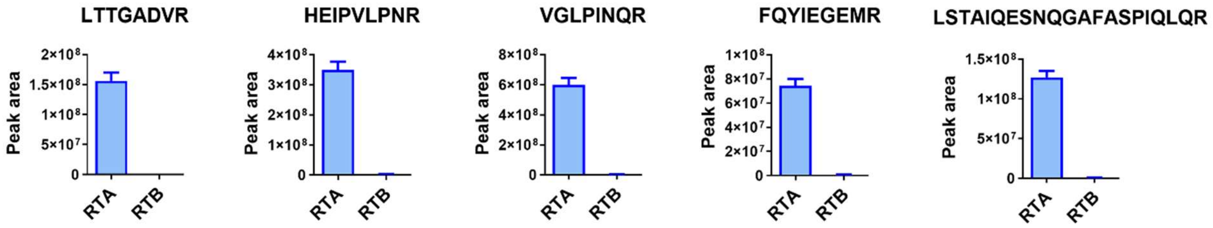


(a)

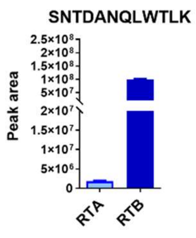


(b)

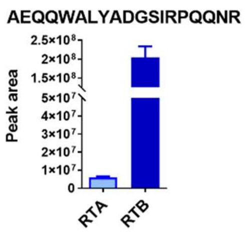


(c)

**Figure S2.** LC-MS/HRMS signals of peptides from **(a)** RTA **(b)** RTB and **(c)** RTB/RCA120 B-chain in commercial RTA or RTB solutions [80]. Peak area is represented as mean ± SD (N=6). Estimated percentage of RTA contamination in the RTB preparation was 0.4 %, based on the peak area of RTA specific peptides (a). Estimated percentage of RTB contamination in the RTA commercial solution was 3.7 %, based on the peak area of RTB (b) and RTB + RCA120 B-chain (c) peptides.

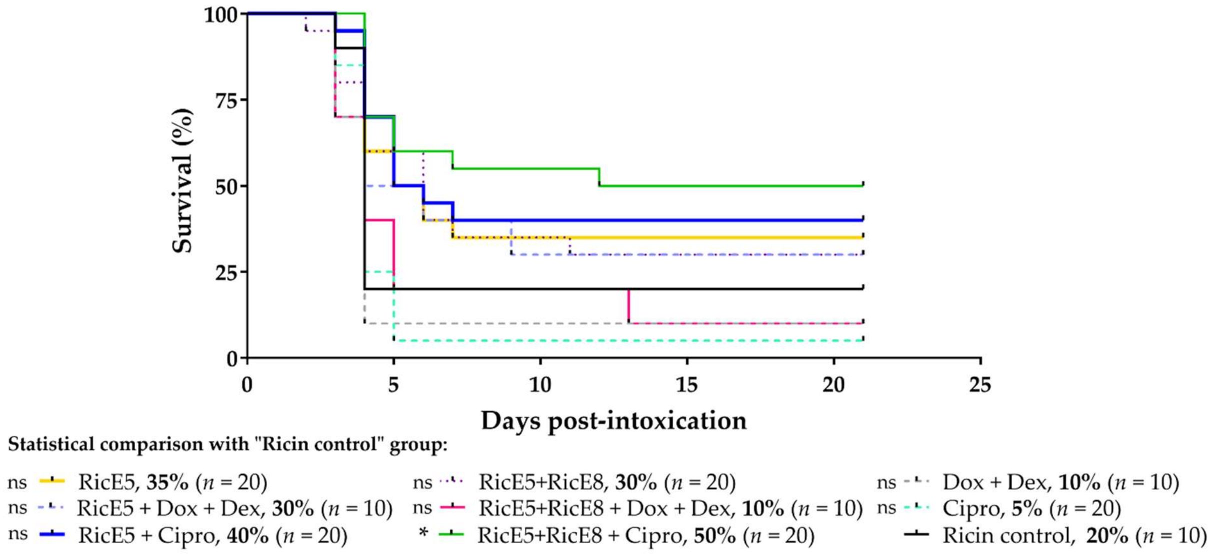


**Figure S3:** Survival curves of mice intoxicated with 5 $LD_{50}$ of ricin D+E and subsequently treated 24h later with 10 mg/kg of mAbs along with anti-inflammatory molecules. Data from two independent experiments are combined. The anti-inflammatory treatments included ciprofloxacin (Cipro) at 200 mg/kg, doxycycline (Dox) at 100 mg/kg and dexamethasone (Dex) at 4 mg/kg. *n* is the number of mice in each group. Survival rates of the treated groups were compared to those of the ricin 5 $LD_{50}$ control group. Statistical analysis: Log-rank (Mantel-Cox). ns: not statistically significant, *: $p < 0.05$; **: $p < 0.01$.

**Table S1.** Summary of the conditions tested for re-exposition. N is the number of independent experiments.

| Treatments after initial exposure | Survival rate after initial exposure | Interval between two intoxications (months) | Number of mice re-exposed | Survival proportions after 2$^{nd}$ exposure |
|---|---|---|---|---|
| RicE5 10 mg/kg, +6h | 92.9% (N= 3) | 10 | 17 | 16/17 |
| | | 12 | 8 | 8/8 |
| RicE5 + RicE8 10 mg/kg, +6h | 89.6% (N = 4) | 10 | 18 | 18/18 |
| | | 12 | 21 | 21/21 |
| RicE5 + RB34 10 mg/kg, +6h | 90% (N = 1) | 10 | 9 | 9/9 |
| RicE8 + 43RCA-G1 10 mg/kg, +6h | 60% (N = 1) | 10 | 6 | 6/6 |
| RicE5 + RB34 + 43RCA-G1 10 mg/kg, +6h | 80% (N = 1) | 10 | 8 | 8/8 |
| RicE5 10 mg/kg,+10h | 30% (N = 1) | 10 | 3 | 3/3 |
| RicE5 + RicE8 10 mg/kg, +10h | 80% (N = 1) | 10 | 8 | 8/8 |
| RicE5 10 mg/kg, +18h | 50% (N = 1) | 10 | 5 | 5/5 |
| RicE5 + RicE8 10 mg/kg, +18h | 40% (N = 1) | 10 | 3 | 3/3 |
| RicE5 10 mg/kg, +24h | 35% (N = 2) | 10 | 4 | 4/4 |
| | | 12 | 3 | 3/3 |
| RicE5 + RicE8 10 mg/kg, +24h | 30% (N = 2) | 10 | 3 | 3/3 |
| | | 12 | 3 | 3/3 |
| RicE5 10 mg/kg, cipro 200 mg/kg, +24h | 40% (N = 2) | 10 | 6 | 6/6 |
| | | 12 | 1 | 1/1 |
| RicE5 + RicE8 10 mg/kg, cipro 200 mg/kg, +24h | 50% (N = 2) | 10 | 4 | 4/4 |
| | | 12 | 6 | 6/6 |
| RicE5 10 mg/kg,, dox 100 mg/kg,, dex 4 mg/kg, +24h | 30% (N = 1) | 12 | 3 | 3/3 |
| RicE5 20 mg/kg, +24h | 44.4% (N = 1) | 12 | 4 | 4/4 |
| RicE5 + RicE8 20 mg/kg, +24h | 50% (N = 1) | 12 | 5 | 5/5 |

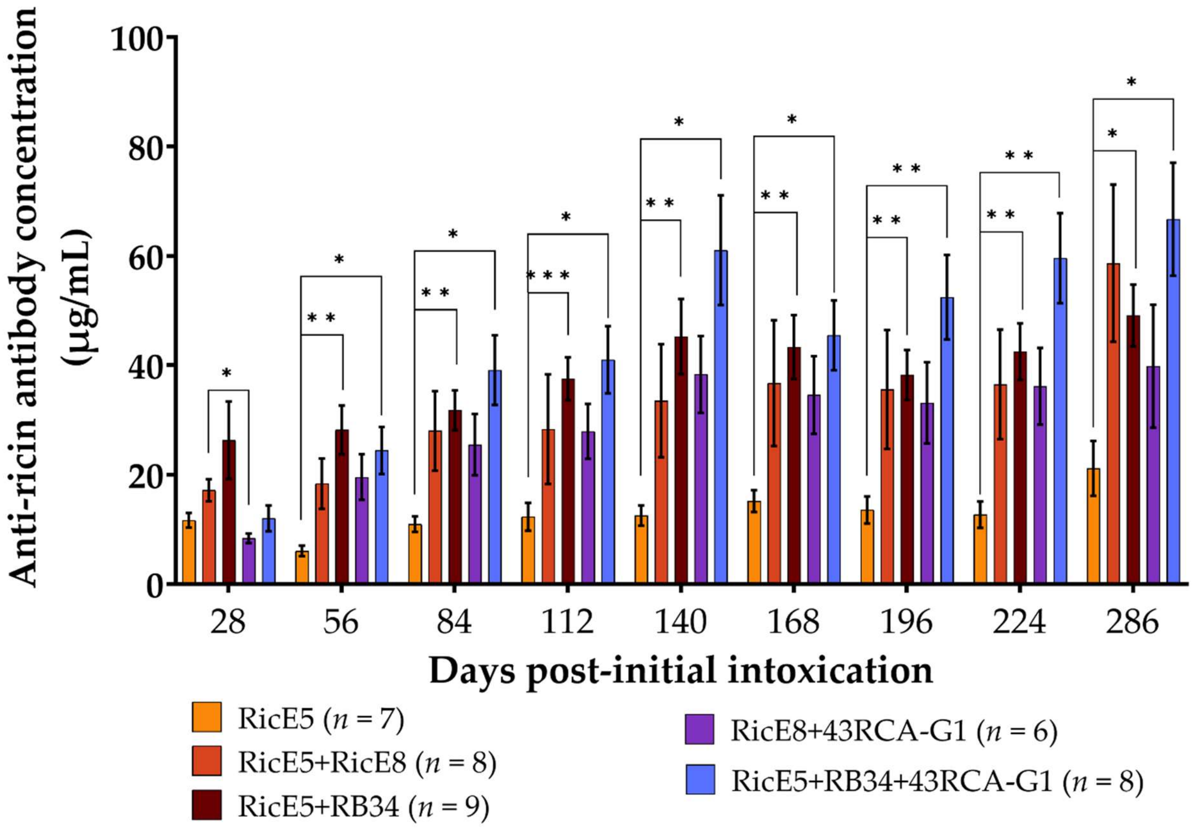


**Figure S4.** Concentration of anti-ricin polyclonal antibodies in mice plasma after first ricin exposure. Each point on the graph represents the mean ± SEM of the circulating anti-ricin polyclonal antibodies in the plasma of treated mice that survived to exposure to 5 $LD_{50}$ of ricin followed by i.v. administration of 10 mg/kg 6h after intoxication (treatments details are provided in the legend; *n* represents the number of surviving mice). Statistical analysis was conducted using a two-way ANOVA with Tukey's multiple comparison test. *: $p < 0.05$, **: $p < 0.01$, ***: $p < 0.001$. Only statistically significant results are shown.
